# The utility of repeated computed tomography to track a foreign body penetrating the esophagus to the level of the thyroid gland

**DOI:** 10.1007/s11282-013-0156-y

**Published:** 2013-10-24

**Authors:** Hai-hong Chen, Ling-Xiang Ruan, Shui-Hong Zhou, Shen-Qing Wang

**Affiliations:** 1 Department of Otolaryngology, The First Affiliated Hospital, College of Medicine, Zhejiang University, Hangzhou, 310003 China; 2 Department of Radiology, The First Affiliated Hospital, College of Medicine, Zhejiang University, Hangzhou, 310003 China

**Keywords:** Foreign body, Migration, Thyroid gland, Computed tomography, Surgery

## Abstract

**Objective:**

Foreign body (FB) ingestion is a common problem in otolaryngology. One uncommon complication of FB ingestion is penetration to the level of the thyroid gland. To our knowledge, only 21 such cases have been reported in the literature. Here, we report a case of an esophageal FB penetrating to the level of the right thyroid gland.

**Case report:**

The patient was a 38-year-old woman in whom an esophageal FB penetrated to the level of the right thyroid gland. We traced the path to the thyroid gland using repeated computed tomography (CT) scans and demonstrated the importance of multiplanar reconstruction in locating the FB and formulating a precise surgical plan.

**Conclusions:**

To our knowledge, this is the first report of repeat CT scans being used to demonstrate the migratory route, over time, of a FB penetrating through the esophagus to the level of the thyroid gland. Our results suggest that multiplanar reconstruction may play a key role in the precise diagnosis of a FB at the level of the thyroid gland and may help surgeons choose the best approach for removal.

## Introduction

Foreign body (FB) ingestion is a common problem in otolaryngology practices [1]. One uncommon complication of FB ingestion is penetration to the level of the thyroid gland and, to our knowledge only 21 such cases have been reported [1–17]. If left untreated, serious complications associated with high risks of morbidity and mortality can develop, such as periesophagitis, periesophageal abscess, mediastinitis, aortoesophageal fistula, innominate esophageal fistula, and carotid rupture [3, 4]. Therefore, prompt diagnosis is essential in the management of perforating FBs [3]. Most FBs can be located using computed tomography (CT). The CT results can then be used to guide the approach toward FB removal, although the path by which esophageal FBs migrate to the level of the thyroid gland remains unclear.

Here, we report a patient in whom an esophageal FB penetrated to the level of the right thyroid gland. We traced the path of the FB via repeated CT scans. Our case demonstrates the importance of multiplanar reconstruction (MPR) in locating an FB and formulating a precise surgical plan.

## Case report

**Fig. 1:**
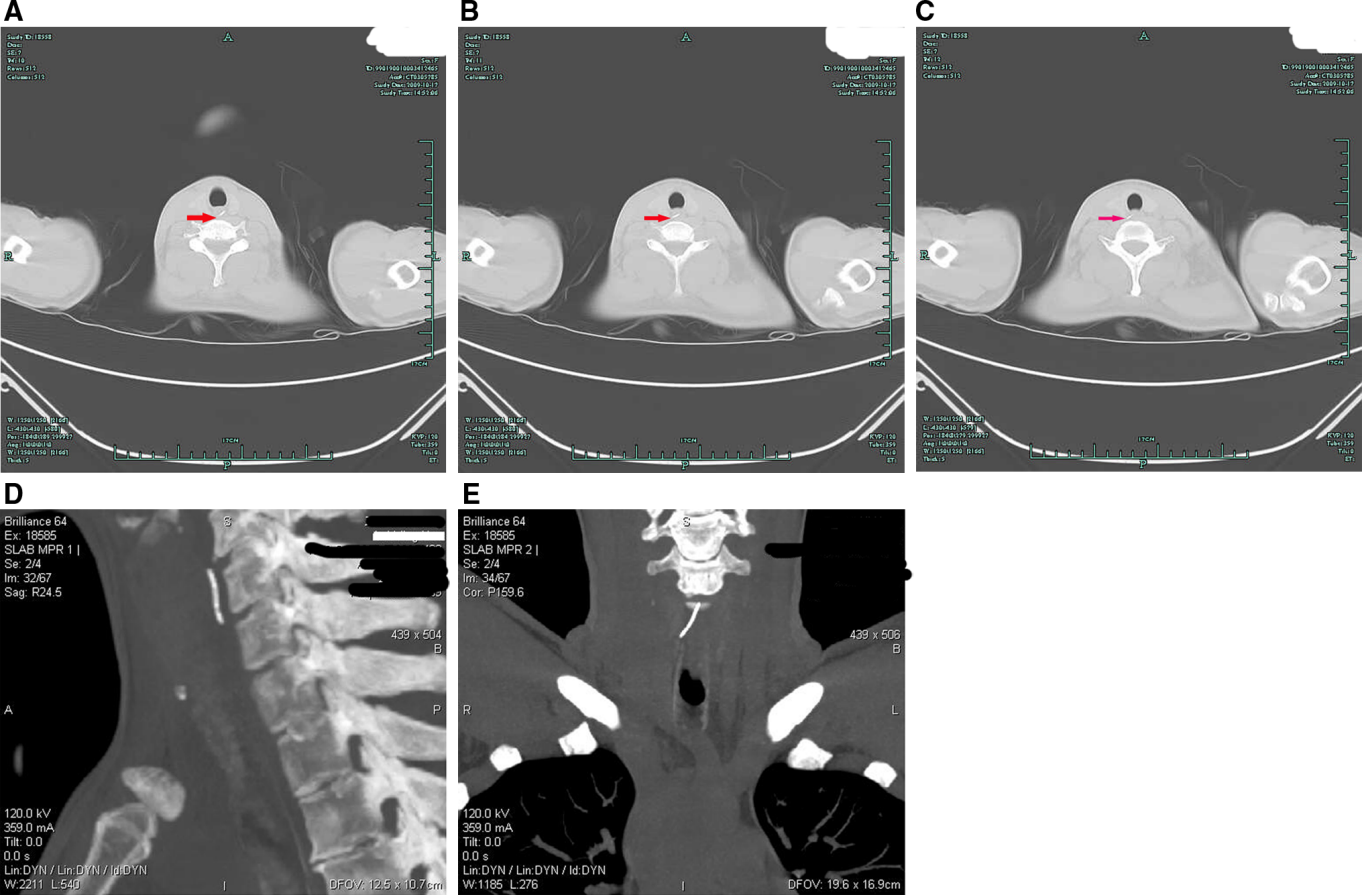
A 38-year-old woman with cervical pain and difficulty swallowing after accidentally ingesting a foreign body (FB) 2 h previously. **a**–**c** Plain transverse images showing the FB in the cervical esophagus (*arrow*), **d** sagittal multiplanar reconstruction (MPR) clearly revealing an oblique radio-opaque linear FB approximately 2.8 cm long located in the cervical esophagus in front of the sixth cervical vertebra (C6), with the long axes of the FB and esophagus making an angle of approximately 13°, **e** coronal MPR revealing that the FB had traversed the right wall of the esophagus

**Fig. 2:**
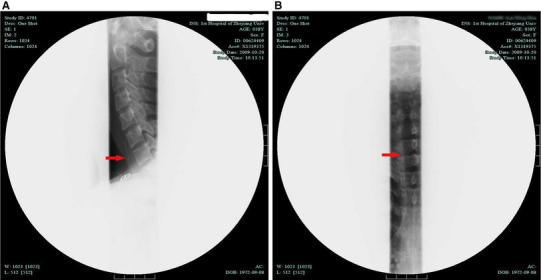
A plain radiograph showing a linear hyperdense area (*arrow*) in front of the C7 and T1 bodies: **a** sagittal image, **b** coronal image

**Fig. 3:**
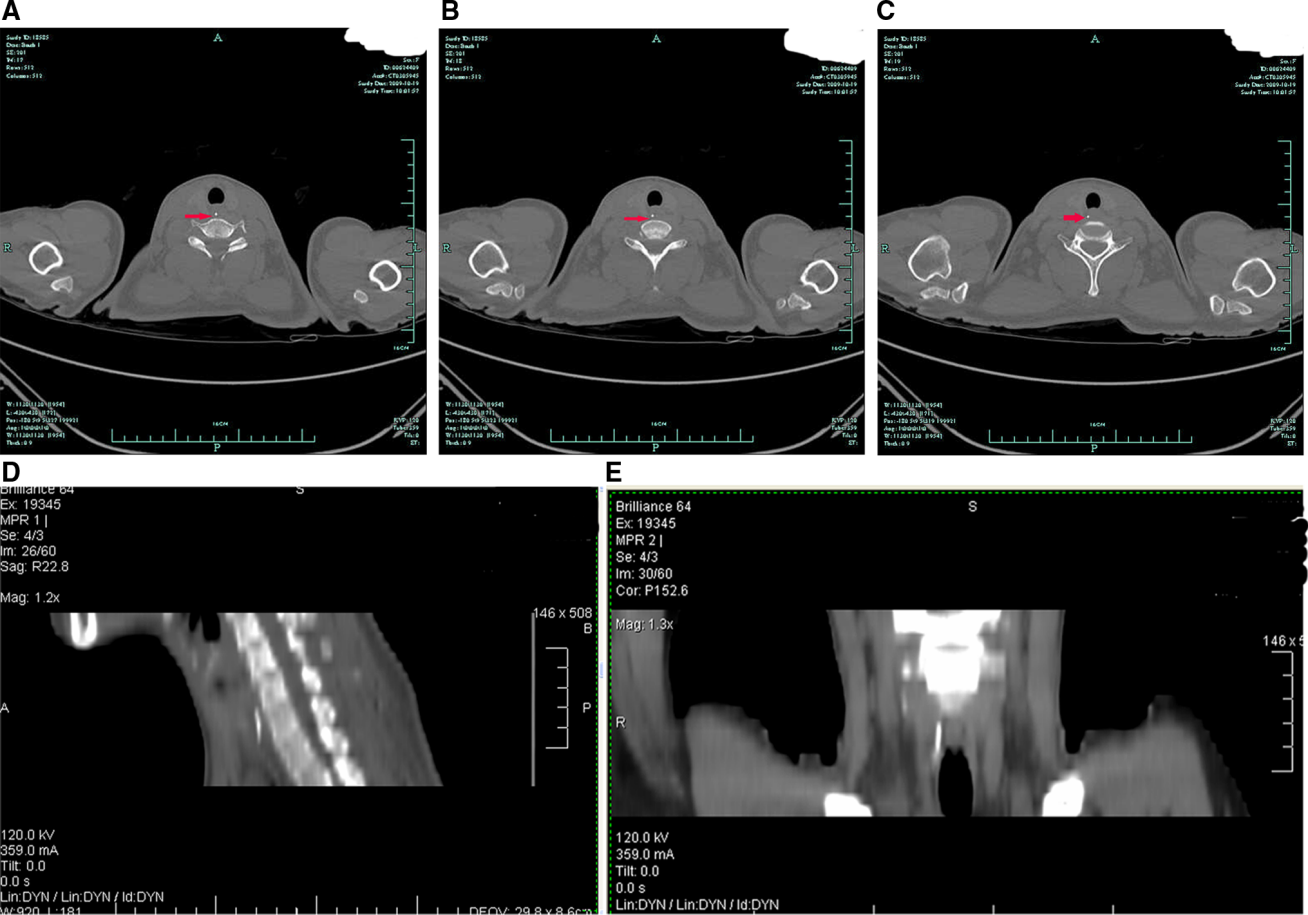
The day after ingestion. A computed tomography (CT) scan revealed a linear hyperdensity located next to the esophageal lumen, indicating that the FB had penetrated the esophageal wall. **a**–**c** Plain transverse images revealing the FB (*arrow*) in the cervical esophagus, **d** sagittal MPR revealing that the FB had moved downward and was now in front of C7, with its long axis paralleling the longitudinal esophageal axis, **e** coronal MPR revealing that the FB had moved into the right paraesophageal adipose space and that its long axis now paralleled the esophageal longitudinal axis

**Fig. 4:**
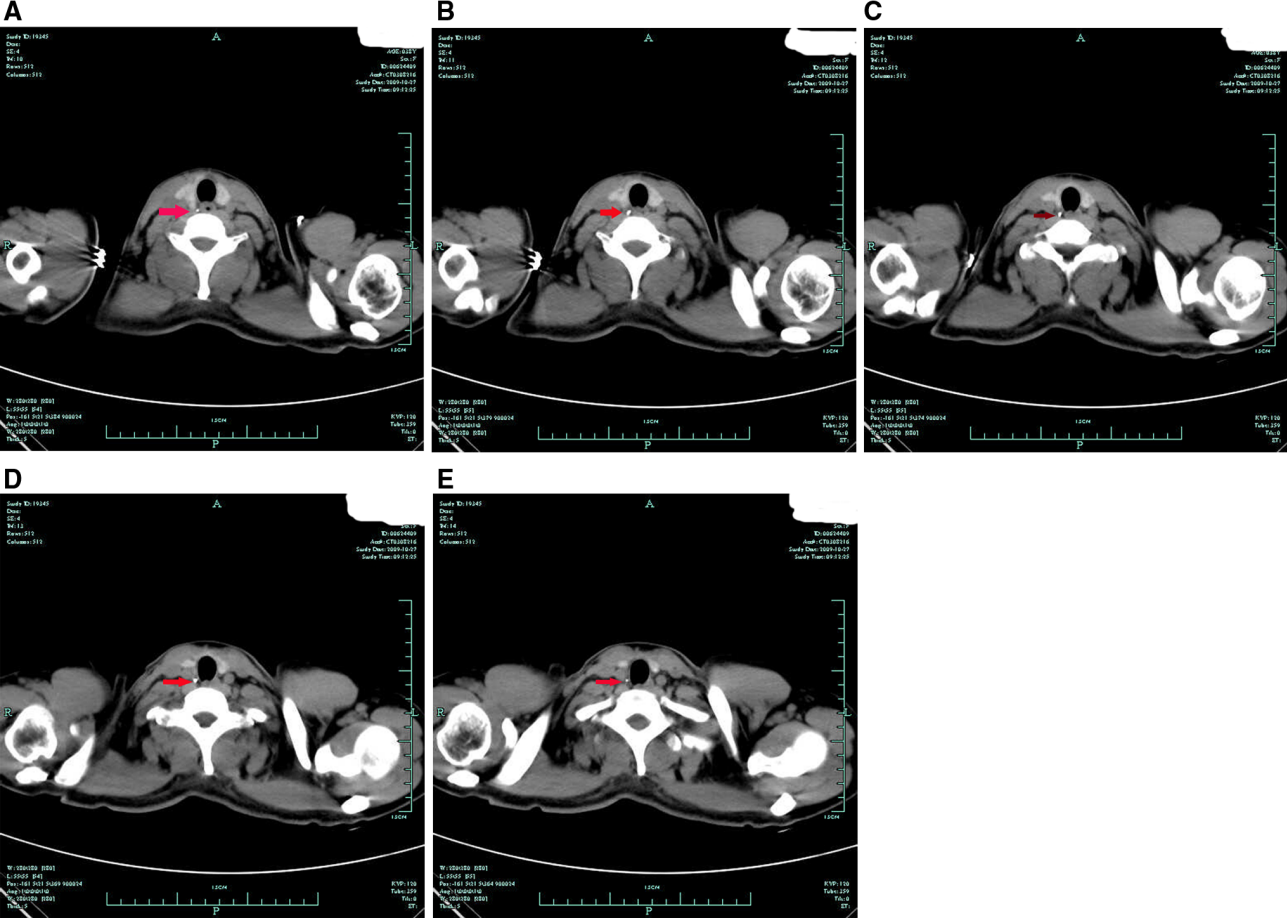
Transverse imaging at 10 days after ingestion revealing a linear hyperdense area (*arrow*) located behind and at the level of the right thyroid gland (**a**–**e**)

**Fig. 5:**
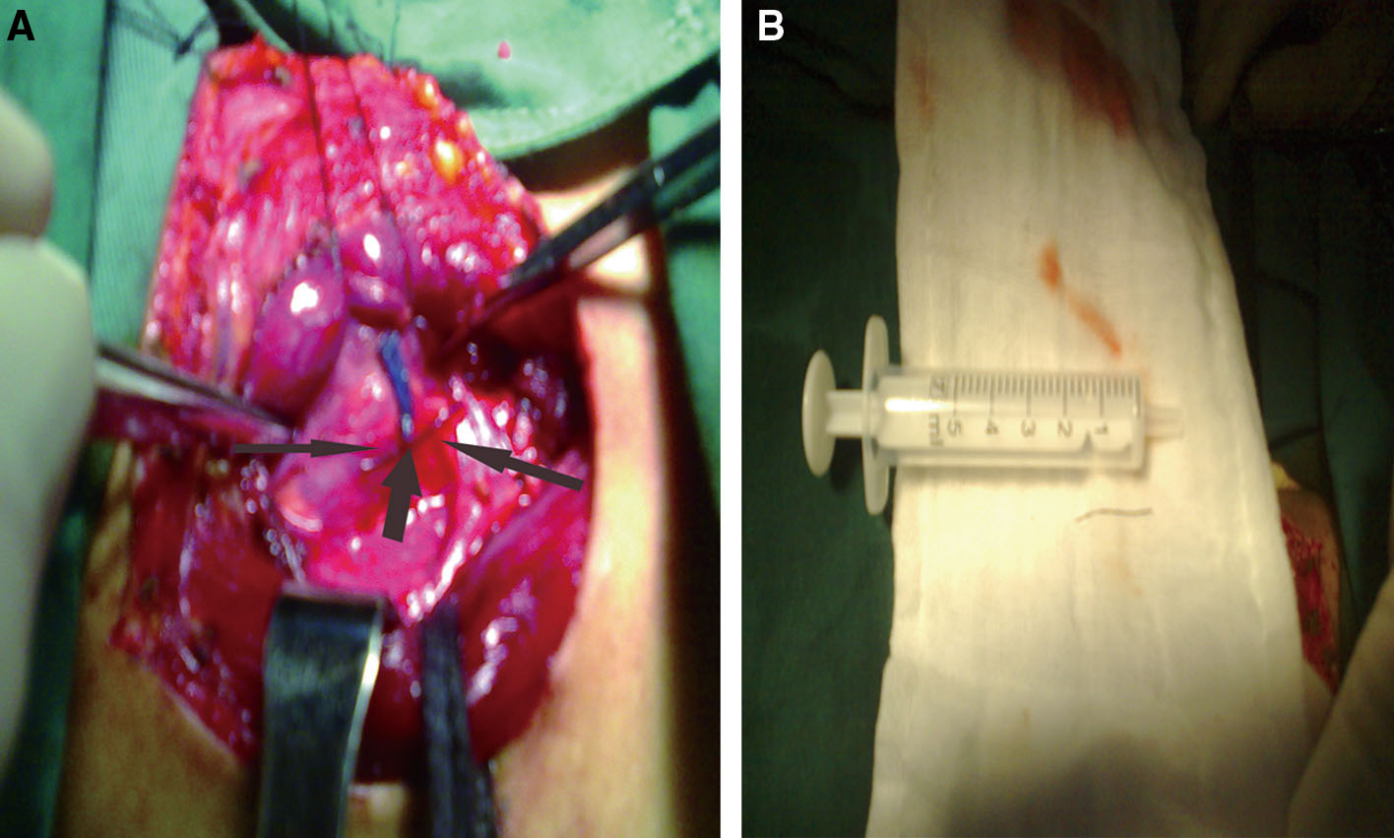
Intraoperative photograph of the FB extraction (**a**). A fine wire (*arrows*), measuring 2.8 cm in length, was identified behind the right thyroid gland (**b**)

A 38-year-old woman presented to the emergency department with a history of sudden painful dysphagia after accidentally ingesting a FB during dinner 2 h earlier. Indirect laryngoscopy showed no FB in the oral cavity, pharynx, or larynx. A CT scan 1 h later revealed a hyperdense FB obliquely traversing the right lateral wall of the esophagus, and MPR clearly showed an approximately 2.8-cm-long oblique radiopaque linear FB in the cervical esophagus, in front of the sixth cervical vertebra (C6). The long axes of the FB and esophagus formed an angle of approximately 13° (Fig. 1). Rigid esophagoscopy was performed under general anesthesia 9 h later, but the FB was not found despite a careful search. The day after ingestion, the patient’s odynophagia was relieved. However, a plain radiograph revealed a linear hyperdense mass in front of the C7 and T1 bodies (Fig. 2). A subsequent CT scan of the neck the next day showed that the FB had penetrated the esophageal wall (Fig. 3a, b, c). MPR demonstrated that the FB had moved into the adipose space in the right paraesophageal area and downward in front of C7. Its long axis now paralleled that of the esophagus (Fig. 3d, e). It was impossible to remove the FB via esophagoscopy, so we suggested removing it using an external approach via lateral neck exploration. However, the patient refused surgery. The patient was given antibiotics for several days, and her symptoms improved greatly; she had no difficulty swallowing. Ten days after ingestion, a CT scan indicated that the FB had migrated to near the right thyroid gland (Fig. 4). The patient underwent an open right neck exploration. Based on the CT findings, the right thyroid gland was exposed and carefully pulled upward; the FB, a fine wire measuring 2.8 cm in length, was located just behind it (Fig. 5). The patient had an uneventful postoperative period.

## Discussion

A migratory esophageal FB is uncommon, and migration of these FBs to the level of thyroid gland is rare. A search of the Medline database using the method set out by Hohman et al. [5] revealed 22 cases of FBs that had migrated to the level of the thyroid gland, including our case (Table 1). Interestingly, all of the patients were adult women, with a mean age of 50.3 (range 21–73) years. The time from ingestion to presentation varied from 2 h to 1 year, with a mean delay in presentation of 20 days; this was longer than the period reported by Hohman et al. [5]. Taken together, these FBs consisted of 16 fish bones, two chicken bones, and four metal wires or bristles; of these, 12 and ten were located at the level of the right thyroid gland and left thyroid gland, respectively, showing an almost equal distribution.

**Table 1:** Cases of foreign body migration into the thyroid gland reported in the literature and our case

Patient no.	Age (years)/gender	Foreign body	Length (cm)	Site	Time to presentation	Plain radiography	Computed tomography	Treatment
Present case	38/Female	Fine wire	2.8	Right TG	2 h	+	+	Open neck exploration without TL
2 [1]	56/Female	Fish bone	2.8	Left TG	1 year		+	Left hemithyroidectomy
3 [2]	69/Female	Fish bone	3.4	Right TG	9 months		+	Open neck exploration without TL
4 [4]	50/Female	Fish bone	4.1	Right TG	1 month	+	+	Open neck exploration without TL
5 [3]	28/Female	Fish bone	NA	Right TG	3 days		+	Total thyroidectomy
6 [3]	56/Female	Fish bone	2.5	Left TG	2 months		+	Right TL
7 [5]	34/Female	Wire bristle	3.0	Right TG	3 days		+	Right TL
8 [6]	26/Female	Wire bristle	NA	Right TG	21 days	+	+	Open neck exploration without TL
9 [7]	61/Female	Fish bone	2.7	Right TL	16 days	–	+	Open neck exploration without TL
10 [8]	21/Female	Wire bristle	2.5	Right TL	N/A	+		Open neck exploration without TL
11 [9]	59/Female	Fish bone	2.0	Left TG	Few days	–	+	Open neck exploration without TL
12 [10]	38/Female	Fish bone	2.0	Left TG	5 days	+	+	Open neck exploration without TL
13 [11, 12]	66/Female	Fish bone	2.5	Left TG	3 days	–	+	Left TL
14 [11, 12]	72/Female	Fish bone	2.5	Left TG	7 days	+	+	Open neck exploration without TL
15 [11]	65/Female	Fish bone	3.5	Left TG	14 days	+	+	Open neck exploration without TL
16 [11]	68/Female	Fish bone	3.0	Right TL	4 days	+	+	Open neck exploration without TL
17 [13, 14]	38/Female	Fish bone	NA	Left TG	Hours	+		Open neck exploration without TL
18 [15]	42/Female	Fish bone	3.5	Left TG	NA	+		Open neck exploration without TL
19 [16]	61/Female	Fish bone	NA	Left TG	21 days	+		Open neck exploration without TL
20 [16]	58/Female	Chicken bone	3.8	Right TL	1 days	+		Open neck exploration without TL
21 [16]	73/Female	Chicken bone	2.5	Right TL	8 days	+		Open neck exploration without TL
22 [17]	28/Male	Fish bone	NA	Right TL	3 days	+	+	Partial right TL

*NA* Not available, *TG* thyroid gland, *TL* thyroid lobectomy

To our knowledge, this is the first report of CT being used to demonstrate the migratory path, over time, of a FB penetrating the esophagus and traveling to the level of the thyroid gland. The FB began to migrate in the esophagus within 10 h and penetrated the esophageal wall into the surrounding adipose space within about 1 day. The contraction of the cricopharyngeal muscle during swallowing probably played a significant role in this process [11]. Given the barrier roles of the anterior (trachea) and posterior (cervical vertebra) regions of the esophageal wall [11], a FB may penetrate only through the lateral wall of the esophagus toward the level of the right or left thyroid gland (no predilection). If untreated, the FB will continue to migrate after swallowing. In our case, the FB took 10 days to travel to the level of the right thyroid gland. The nature of the FB and the angle between the FB and esophageal wall may also play a role in the migration process. All of the cases reported to date involved hard, pointed FBs [1–17], with the FBs having a mean length of 2.8 (2.0–4.1) cm. Our patient swallowed a fine, pointed wire. The nature of the FB may facilitate transmural esophageal penetration and subsequent migration to the level of the thyroid gland [11]. Al-Sebeih et al. [17] suggested that the more horizontally oriented and sharper the FB is, the higher the risk of penetration. However, we found that the FB migrated easily with a small acute angle between the long axes of the FB and esophagus. Of course, we cannot rule out the possibility that pushing on the rigid esophagoscope facilitated the migration. If a FB is not removed, it will continue to migrate and may cause serious complications, such as aortoesophageal fistula or carotid rupture [3, 8]. Furthermore, a FB can introduce bacteria into the soft tissues of the neck, causing suppurative complications such as parapharyngeal or retropharyngeal abscesses and mediastinitis [3, 8]. Fortunately, no serious complications occurred in our case.

For the above-mentioned reasons, it is very important to remove a FB in a timely manner. However, it is difficult to locate a migrated short fine wire in the neck via an open approach. The main difficulty is localizing the FB in the soft tissues, after which removal is usually simple. Traditional approaches to locate an impacted FB generally include using plain films of the neck and chest, barium swallow, and direct esophagoscopic examination. A plain radiograph may also indicate the level of the FB and may be useful in a follow-up context, as in the case of our patient. However, plain radiography usually affords only poor sensitivity when the FB is a fish or chicken bone because bones are rarely visible on radiographs [18, 19]. A plain radiograph is therefore suitable only for searching for the more radiodense FBs.

Barium swallows have yielded different results in different cases [20, 21]. Many endoscopists emphasize their negative features. Mosca et al. [21] reported that barium involves a risk of aspiration, can delay an emergency endoscopic procedure, and can complicate removal of a FB by interfering with endoscopic visualization. Therefore, in some hospitals, the patient is sent immediately for esophagoscopy, without a diagnostic imaging assessment [22]. Esophagoscopy, even those using flexible endoscopes, is an invasive technique with a potential morbidity risk [22]. CT is an important diagnostic technique in cases of migrating esophageal FBs. Although a plain radiograph may show the level of the FB, we found that repeated CT scans showed the migratory path, over time, of the FB more clearly and that MPR (commonly employed in the evaluation of various diseases, such as fractures of the skull base [23]) may be helpful when used to diagnose a FB at the level of the thyroid gland. Using axial and coronal reconstruction, MPR can show the shape, size, length, location, and direction of the FB, thereby providing important information to guide the surgical approach. Using various imaging angles in multiple series of images can reveal the anatomical relationship between an impacted FB and vital structures of the head and neck, such as the trachea, cervical vertebrae, thyroid gland, and arteries of the neck. These data can help surgeons choose the best approach to remove the FB and reduce surgical complications. We exposed the right thyroid gland directly through a lateral neck incision and removed the fine wire successfully.

In conclusion, to our knowledge, this is the first demonstration of the migratory trace and time of a FB penetrating through the esophagus to the level of the thyroid gland using repeated CT. We also demonstrated that MPR can play a key role in the diagnosis of a FB at the level of the thyroid gland and help surgeons choose the best approach to remove the FB and reduce surgical complications.
